# Future Healthcare Workers and Ecopharmacovigilance: Where Do We Stand?

**DOI:** 10.3390/pharmacy12050146

**Published:** 2024-09-26

**Authors:** Toni Durdov, Ana Šešelja Perišin, Nikolina Škaro, Josipa Bukić, Dario Leskur, Darko Modun, Joško Božić, Marjeta Grgas, Doris Rušić

**Affiliations:** 1 Department of Pharmacy, University of Split School of Medicine, Soltanska 2A, 21000 Split, Croatianina.skaro3@gmail.com (N.Š.); jbukic@mefst.hr (J.B.); dleskur@mefst.hr (D.L.); dmodun@mefst.hr (D.M.); ma71906@mefst.hr (M.G.); drusic@mefst.hr (D.R.); 2 Department of Laboratory Medicine and Pharmacy, Faculty of Medicine, Josip Juraj Strossmayer University of Osijek, Josipa Huttlera 4, 31000 Osijek, Croatia; 3 Department of Pathophysiology, University of Split School of Medicine, Soltanska 2A, 21000 Split, Croatia; jbozic@mefst.hr

**Keywords:** ecopharmacovigilance, drugs disposal, antibiotic resistance, eco-directed sustainable prescribing, unused drugs

## Abstract

With the rapid development of the pharmaceutical industry and constant growth of drug usage, ecopharmacovigilance (EPV) has emerged as a way of coping with and minimizing the effects that drugs have on the environment. EPV concerns and describes unwanted effects that the use of a specific drug could have on the environment. The US, EU and Cananda are the improving position of EPV, both in legislation and practice. EPV requires further development as previous studies have shown that neither the general population nor healthcare professionals have enough knowledge about the subject. Improving awareness and knowledge about this topic is a key task for the future of EPV. The main objective was to determine students’ level of knowledge about ecopharmacovigilance and to examine ways of storing and disposing of unused and expired drugs. Students’ knowledge and habits were examined by a previously published survey. The survey contains twenty questions divided into three parts and the possibility of writing an additional note. There was no difference in the level of knowledge between the students of different studies. Also, students who had a family member working as healthcare professional did not show a higher level of knowledge compared to the others. Pharmacy students had a greater intention to educate their environment about EPV when compared to students of the other studies. This is in the line with a previous study which showed that the general public expects that pharmacists and physicians educate them about EPV. Medicine and dental medicine students will become prescribers after finishing their studies, and as such, they should be informed about eco-directed sustainable prescribing (EDSP) as part of an EPV strategy. More than half of the participants reported good adherence to prescribers’ instruction, which decreased the amount of unused drugs. Most of the students found that the drug expiration date was legible, but they did not check it often. In comparison with similar studies, Croatian students had more knowledge and better practices concerning EPV and drug disposal. Structured learning strategies and curriculum implementation for EPV are much needed for further raising awareness about the subject among healthcare professionals and the public.

## 1. Introduction

Climate change, environment-care and ecology are everyday topics. This is entirely deserved given the speed at which changes are happening in our surroundings and the consequences they entail [1]. The negative impact of humans on existing ecosystems is not questionable but measurable, tangible, and, above all, long-lasting [2]. Often, in an attempt to improve the quality and duration of life, people forget or neglect other organisms in their environment. An excellent example of this is the development and application of medications, which we know contribute to the longevity and quality of life. Medications are the foundation of today’s medical care, a tool with which we fight and prevent various diseases. The availability of necessary medication is the right of every human being [3].

In recent years, the pharmaceutical industry has increased its focus on the impact of pharmaceutical products in the environment. Medications can enter the environment at all stages of their life cycle, from production to consumption and waste disposal [4]. In the environment, they often manifest some unwanted and unforeseen effects. While the adverse effects of drugs in humans have been classified and investigated since the thalidomide crisis of the 1960s and categorized under pharmacovigilance, until recently, there has been no global discussion of these effects on the environment [5,6]. However, the harmful effects of drugs, their metabolites, and APIs (active pharmaceutical ingredients) are ubiquitous and increasingly well researched and documented.

In the context of all the above, EPV (ecopharmacovigilance) has emerged. EPV was first mentioned by G.P. Velo in 2007 [7]. The World Health Organization (WHO) describes pharmacovigilance as “the science and activities relating to the detection, assessment understanding and prevention of adverse effects or any other possible drug related problems” [8]. When talking about EPV, it is only reasonable to implement that definition to the environment so that EPV can “describe the science and activities associated with the detection, evaluation, understanding and prevention of adverse effects of pharmaceuticals in the environment” [9]. EPV is used to describe the unforeseen consequences that APIs can have on the environment [5]. EPV promotes sustainability, rational consumption, the disposal of unused medicines, and the minimization of residues in production [10]. EPV strategies should be targeted for drugs that have already been proven to have adverse effects on the environment. Serious pollution problems and high-risk areas should be the focus [11]. The implementation of EPV in practice has already begun. In the EU, an ERA (environmental risk assessment) is required for the launch of a drug on the market [9]. The EMA (European Medicines Agency) implemented guidelines on ERAs, which state that every medicinal product for human use should have an ERA prior to its market release. It also states that an ERA should be updated for every major change regarding the product (indication, way of application, etc.) [12]. The US, EU and Canada are world leaders in EPV legislation [4]. Some EU pharmaceutical companies, such as AstraZeneca, have developed ERMPs (environmental risk management plans) for easier environmental impact management [13].

The global EPV system has a lot of room for improvement. Low-income countries often do not have any strategy for collecting and disposing pharmaceutical waste, and even when they have one, it is mostly reserved for larger facilities and manufacturers, not available to common households. EU countries should have a clear unused and unwanted drug collection policy. In some countries (including Croatia), pharmacies are obliged to collect pharmaceutical waste, other countries organize said collection through non-profit agencies (Portugal) and there are still a number of countries that do not have clear guidelines about said problem (Poland and Romania). In the US, the Drug Enforcement Administration (DEA) published the Final Rule on the Disposal of Controlled Substances in 2014, which gives patients the option to either hand over unused drags at take-back events or to mail them to an authorized collector with appropriate labeling [14].

Clearer roles and the division of labor between regulatory agencies, an organized system of technical support, education strategies, and financial support are needed [15]. EPV combined with a drug take-back program and further engagement from drug manufacturers both logistically and financially is needed to properly address the pharmaceutical waste issue [16]. The results of various studies on the perception of EPV among healthcare professionals and the general population have shown the need for the implementation of a clear education strategy on this topic [15].

One study conducted in China showed that general population expects education about pharmacovigilance from healthcare professionals, preferably pharmacists and physicians [17]. Pharmacists could play an important role in the education of the elderly, while the younger population can be reached through social media [14]. Also, the Pharmaceutical Committee of the European Commission, Health and Food Safety Directorate—General in its document “Revised mandate of the ad-hoc working group to focus on the EU strategic Approach on pharmaceuticals in the environment” highlighted the need for the education of healthcare professionals in this subject: “Explore, in cooperation with relevant stakeholders, how environmental aspects could become part of medical training and professional development programs” [18]. There are already examples of the EPV being implemented into the formal education of future health workers. At the University of Groningen pharmacy students participated in a module called “Reducing Pharmaceuticals in Water” [19]. In Spain, there is even a postgraduate course on pharmaceutical pollution and four EU universities started a 2-year study program called “Sustainable Drug Discovery” [20,21].

With formal education on EPV already being integrated into the curricula at several universities, and numerous previous claims highlighting the need for a clearer and more structured educational strategy, this study aims to assess the current knowledge level of students regarding this important topic. These students, who will hopefully become healthcare professionals in a few years, are particularly significant to the study as they will soon be expected not only to apply this knowledge in their professional practice but also to educate the general population on the EPV topic. Healthcare professionals, especially prescribers and dispensers, play a crucial role in promoting a rational prescribing system that ensures the safe and effective use of pharmaceuticals, with a particular emphasis on the responsibilities of the prescriber. It is equally important for future healthcare professionals to guide and educate their senior colleagues, as this field is rapidly evolving. The importance of understanding EPV cannot be overstated, given its impact on public health and environmental sustainability.

The primary objective of this study is to contribute to the development of a comprehensive educational strategy on EPV in Croatia. By identifying gaps in student knowledge and examining their behaviors and attitudes towards the use and disposal of medicines, this research seeks to establish a baseline from which educational interventions can be designed and implemented. Understanding how students currently manage pharmaceutical products in their personal lives provides valuable insights and highlights areas where further education and training are needed. In summary, by assessing both the knowledge and practices of students regarding EPV, this study aims to set the groundwork for a formal educational strategy about EPV that will ensure that healthcare professionals serve as both enforcers of the EPV and educators about the topic.

## 2. Materials and Methods

The University of Split School of Medicine, consists of 4 programs: Pharmacy, Dental Medicine, Medical Studies and Medical Studies in English. All students enrolled at the University of Split School of Medicine during the academic year 2022/2023 were considered eligible for participation in the study, except for those in the Medical Studies in English program. As the questionnaire was in Croatian, it was not administered to students in the Medical Studies in English program. To administer the questionnaire to the Medical Studies in English, it needed to be distributed in the English language, and most of Croatian students were fluent in English. The study was completely anonymous and voluntary, so by distributing questionnaire only in Croatian language, study tried to avoid possibility of double participation of Croatian students. Notably, EPV is not included in the curriculum of any of programs at the University of Split School of Medicine (Supplementary Materials). 

A cross-sectional study was carried out to assess the knowledge and perceptions of students at the University of Split School of Medicine, regarding EPV. This study utilized an anonymous questionnaire administered during May and June of 2023. Data collection was conducted through an online survey using Google Forms. Students of medicine, dental medicine and pharmacy received the survey link via their respective year representatives. As mentioned in the questionnaire’s introduction section, participation in the study was voluntary and anonymous, and students received no incentives for their participation. The research protocol received approval from the Ethics Committee of the University of Split School of Medicine.

The survey used in the study was based on research completed by Jha et al. [22]. It was subsequently translated from English to Croatian and then back into English by a native speaker. To guarantee its suitability for students, the original questionnaire was reviewed by four professors from diverse fields at the University of Split School of Medicine (USSM). The questionnaire is divided into three sections. The first section asked about participants’ demographic information. The next section contained questions about medicine storage and disposal, with an emphasis only on medicinal products and not on dietary supplements. In this section, participants’ knowledge was evaluated, as correct answers were scored as ‘1’ and incorrect answers as ‘0’. The last section of the questionnaire contained questions about participants’ habits on medicinal product storage and their disposal. It also included a space where students could leave additional feedback regarding unused drugs in their respective homes.

The first component collects sociodemographic information about the students, including their field of study, academic year, gender, age, and place of living. Multiple-choice questions with one correct answer make the second component of the survey. It assesses students’ understanding of how to store unused and expired drugs, as well as their environmental impact. It also investigates the education obtained by healthcare professionals and the general public regarding safe disposal techniques for these medications. The final component of the questionnaire looks into the students’ habits, such as how often they take medications, their tendency to examine dates of expiration, and how they handle expired medicine.

The study results were reported as both whole numbers and percentages. The questionnaire data were exported to a Microsoft Office Excel 2021 spreadsheet, where a descriptive statistical analysis was carried out. Statistical analysis was carried out with the MedCalc program (v.11.5.1.0, MedCalc Software, Ostend, Belgium), which used the Chi-square test for categorical variables. Where Chi-square was not appropriate due to its limitation, Fischer’s exact test was used. The normality of data distribution was determined using the Kolmogorov–Smirnov test. *p*-values < 0.05 were considered statistically significant. Additionally, knowledge scores were calculated by summing relevant responses and examined using the Kruskall–Wallis test. The results were reported as the median and interquartile range. The highest possible score was set at 11.

## 3. Results

The study included 80 students from the University of Split School of Medicine. All of the participants who had enrolled in the study completed it accordingly. Table 1 shows the demographic characteristics of the participating students. A large percentage of students were female (83.7%), with only 16.3% being male. Half of the students were from the field of medicine, with the smallest percentage being first- and fourth-year students.

When asked if they had used medication in the previous six months, the vast majority of students answered affirmatively: 24 pharmacy students (88.9%), 35 medical students (89.7%), and 11 dental medicine students (78.6%).

In terms of where students reside, 46 (57.5%) reported living with family, 30 (37.5%) in private accommodation, and 4 (5.0%) in university dormitories.

Table 2 shows that students almost never buy medications without a prescription, but they are inclined towards self-medication. Specifically, 33.7% of students stated that they engage in self-medication constantly, and 35.0% do so often.

Students were also asked “What do you do with any quantity of purchased medicine remaining unused at your home/hostel?” Most of the students stated that they keep drugs at home until they expire (41 student, 51.20%). In addition, 21 of them (26.20%) said that they threw their drugs into the garbage, and 16 of them (20.00%) returned the drugs to the pharmacy.

Students’ answers regarding whether medicine dosage forms have dates clearly and legibly indicated are shown in Table 3. In general, 68.7% of students gave an affirmative response, and there was no discernible variation between study fields (*p* = 0.069).

Most students—37 or 36.63% (42.90% of dental medicine students, 37.0% of pharmacy students and 53.80% of medicine students)—cited forgetfulness as the barrier preventing them or their family members from using prescription drugs. Furthermore, 20 (19.8%) students (35.70% of dental medicine students, 33.30% of pharmacy students and 15.40% of medicine students) mentioned unfavorable drug side effects, 9 (8.91%) students (21.40% of dental medicine students, 3.70% of pharmacy students and 12.80% of medicine students) mentioned medication ineffectiveness, and 35 (34.65%) students (57.10% of dental medicine students, 44.4% of pharmacy students and 41.00% of medicine students) cited improvements in their health as justifications. When asked if students have ever educated their friends/family members about safe medication disposal, 14 (51.9%) pharmacy students, 8 (20.5%) medical students, and 3 (21.4%) dental medicine students responded affirmatively. Pharmacy students had the highest percentage of affirmative responses, showing a significant difference among the fields of study (*p* = 0.018).

Table 4 shows the percentage of the correct answers in the second section of the questionnaire by the field of study. Total knowledge was shown as the total number of the correct answers in the questionnaire. The results of the Kruskal–Wallis test comparing total knowledge scores by the field of study (median and interquartile range) were as follows: pharmacy 8 (7.25–9), medicine 9 (7–9), and dental medicine 9 (8–9). The *p*-value was 0.534, indicating that there was no significant difference in knowledge among students based on their field of study. Additionally, knowledge was compared among students based on whether they have a family member (father, mother, or sibling) in healthcare. Participants with a healthcare professional in the immediate family achieved a score of 8 (7–10), while participants without a family member in healthcare achieved a score of 9 (7–10). There was no significant difference in knowledge between the groups (*p* = 0.254). Table 4 shows how many students of respected study answered a specific question correctly. As seen, only question number 1 had a significantly different proportion of correct answers as pharmacy students had the most correct answers out of the three groups.

The comments in Table 5 reflect students’ concerns about rational medication use, the proper disposal of expired medications, and the need for comprehensive education for both healthcare professionals and the general public to raise awareness about the implications of unused medications at home.

## 4. Discussion

This study found no significant difference in the knowledge about EPV between students at the University of Split School of Medicine, based on their field of study. Also, it showed no significant differences in knowledge between students who had healthcare professionals in their family and those who did not. It was expected that students whose family members were health professionals would have had a higher level of knowledge about EPV when compared to the other students. A higher level of knowledge was also expected from pharmacy students, considering their study is focused mostly on drugs and the disposal of expired and unused drugs is defined as a key task of pharmacists [23].

It is interesting to note that pharmacy students more often than students of other studies tend to educate their family and friends about the correct ways to dispose expired medications. Research on the general population has also shown positive attitudes on this topic, but again, with a low level of knowledge among respondents. Respondents were aware of the harmful consequences of the improper disposal of unused medicines and expired medicines but did not know how to dispose of them properly [17,24]. European studies had similar results. In most of the countries that examined this problem, only a quarter of the study participants returns expired drugs to the pharmacy [25]. Similar research was conducted in Croatia. It showed that collecting unused medicines was below the European average and the service of collecting unused medicines had a lot of oversights, for example, the lack of privacy when disposing of medicines, the financial burden for pharmacies, etc. [23].

As it was stated before, a Chinese study showed that the general public expects pharmacists and physicians to educate them about EPV [17]. One of our participants also stated something similar: “Pharmacies should educate people more about disposing of expired medications.” It is promising that more than half of the pharmacy students have already educated their close ones about some aspects of this topic. Pharmacists are one of the most important sources of information regarding medications and generally public health [26]. Their availability and knowledge make them the potential focal point of an EPV education strategy. Another research study has shown that pharmacists have very little knowledge about EPV. The mentioned study showed that only 20% of them had read any literature on the subject [27]. This highlights the need to educate healthcare professionals properly and continuously before they proceed to educate the general population. A recent study in Saudi Arabia is more encouraging. It states that around three quarters of community pharmacists returned unused medication to distributors, around 15% disposed of it in a medicine bin and the rest, approx. 10%, disposed of it incorrectly [28].

After completing their studies, medicine and dental medicine students will become prescribers, and their role in promoting EPV will become significant. Prescribers should also play a significant role in implementing EPV strategies [5]. EDSP (eco-directed sustainable prescribing) consists of two segments: prescribing low doses of drugs and prescribing drugs with an environmentally friendly excretion profile [29]. Information about the excretion profiles of drugs are not always the most available ones. For instance, only Sweden of all European countries has an official database that maps the environmental impact of all drugs used in the country [30]. Research in China has shown that prescribers have positive attitudes towards EPV and EDSP but currently do not consider the environmental impact of drugs when prescribing, while research in India has shown that prescribers often do not adhere to guidelines proposed by relevant institutions [29,31]. Although medicine and dental medicine students showed the same level of knowledge about this topic as pharmacy students, they are less inclined to educate others about it. Since EDSP is not included in any subjects’ curriculum at the School of Medicine, it would be beneficial to implement learning about their role in EPV in their respective studies, and it is encouraging that some of them share this view: “I believe education on this topic is necessary, both for the general population and healthcare professionals, because nobody has clearly informed us about such issues personally.”

The majority of students (61.2%) answered the question “How often do you take your medications based on advice from physician/pharmacist?” with “Always” or “Often”. This is especially interesting in the context of using antibiotics since they are one of the most prescribed drugs in Croatia [32]. A particular danger is the presence of antibiotics in the environment, which has been proven in various studies all over the world [33,34,35,36]. Antibiotic resistance is becoming an increasingly common obstacle to treatment, and the growing concentrations of antibiotics in the environment further contribute to the emergence of resistance. This makes antibiotic resistance one of the main topics of EPV [37]. Adherence to the instructions given by prescribers vastly decreases the amount of the medical waste that is produced [25]. It is interesting that a large part of the participants (approx. 40%) do not often adhere to the prescribers’ instructions, which is particularly indicative in the context of they themselves becoming prescribers in a few years. Participants in this study also stated that their families, themselves included, tend to adhere to drug therapy, even in cases where multiple drugs are prescribed to them. However, in future research, it would be of value to expand the questionnaire with questions such as “How often do you take your medications based on advice/advertisement from the internet, TV etc.” to obtain more insight on what drives individuals to choose certain medications or demand certain medications from their physicians that they may not even need and that may end up in communal waste.

Drug production, prescription and consumption is soaring worldwide [25]. Drugs are becoming more available to patients directly. The majority of our participants also stated they practice self-medication for milder diseases, and also, they stop treatment when they are feeling better, which is often before the whole drug package has been used. This often leads to drugs expiring. As one of the participants said, “Unused medications in my household are usually some over-the-counter pain relievers, rarely/never antibiotics or other nonsteroidal anti-inflammatory drugs.”. Previous research has shown that expired drugs lose their therapeutic effect and can cause side effects both to patients and the environment. Most people will throw expired medicine in common garbage cans [25,38]. Most of the students said that the drug expiration date is clearly and legibly written on packages, but less than half of them said that they check the expiration dates of medicines they keep at home more frequently than “Sometimes”. Patients in previous studies often reported that the drug expiration date is not clearly legible, but students that participated in this study are on their way to becoming healthcare professionals and they are coming across medicine packages through their education, and they are probably more prepared to find wanted information about any kind of medicine [39]. 

A similar research study to this one was conducted in Nepal, where medical and dental students were surveyed on the topic of medical waste disposal and leftover medicines. Students do not cover these topics in the curriculum. It was found that only 10% of students disposed of unused and expired medicines properly [22]. Another study was conducted in Ethiopia among pharmacy students. Just over half of the questioned students were aware of the negative impact of improper drug disposal on the environment, and only 20% of the students had heard of the term EPV. Although the majority of the students showed a positive attitude towards EPV, more than 60% of them threw unused medications in the trash [40]. A Nigerian study from 2023 compared knowledge about drug disposal between students of various biomedicine studies such as pharmacy, medical laboratory science, medicine, nursing, etc. The aforementioned study did not find significant differences in the knowledge about drug disposal between the participants based on the field of study, as was the case in our study [41]. In these studies, different questionnaires were used, so the results are not directly comparable, but Croatian students showed an admirable level of knowledge about this topic as was expected due to differences in socio-economic status and healthcare system organization. In comparison with a high-income country, a survey in Saudi Arabia showed that 67% of pharmacy students considered returning unused drugs to the pharmacy as the best practice, but it was unclear how many of them actually did it, while our study showed that 20% of students followed this practice [42]. Another Saudi Arabian study showed that 78.9% of the pharmacy students included threw away expired medicine in the garbage or flushed it in the toilet or the sink. Only around 7% returned expired drugs to the pharmacy, which was a significantly lower percentage compared to our study [43].

Students at the University of Split School of Medicine, further highlighted the need for the constant education of healthcare professionals. Even though their knowledge level was satisfactory, they did not follow it up in practice, with only a small number of them disposing unused medicine in the correct way. A great example of the implementation of EPV into the curriculum of biomedical studies is a project at the University of Groningen, where third-year master’s students in Pharmacy were introduced to an educational module called “Reducing Pharmaceuticals in Water”. Students learned about pharmaceutical pollution and ways to prevent it from different perspectives (pharmacists, prescribers, legislation, etc.). The module was interactive and consisted of multiple videos and cases that students solved. With this type of education, we could encourage students to implement the theoretical knowledge they have [19]. 

As there is no mention of EPV in the curriculum of any biomedicine study in Croatia, the next steps would be to use the results that this study gathered to create a proper EPV educational strategy. This study assessed the baseline knowledge of students about drug disposal and their general behavior regarding using and disposing of medicine. The collected data showed that prior to implementing EPV-related subjects and diving into the more complex examples of the relationship of medications and the environment, education should focus on improving the personal habits and preferences of biomedicine students in context of their drug usage and disposal. Since only one-quarter of students had a habit of returning unused drugs to the pharmacy and students did not show a strong commitment to further educate their friends and families, it is crucial to emphasize the importance of setting their own example that other people can follow as stated before when numerous studies identified healthcare professionals as designated EPV educators.

The biggest limitation of this study is the small number of participants, followed by the narrow time window in which it was carried out. Given that it involves a survey questionnaire, one limitation is response bias, where answers may not always reflect the actual behavior of the respondents. Another significant limitation of the questionnaire is the reliance on the participants’ memory. Respondents may not accurately recall past events, behaviors, or experiences, leading to potential inaccuracies in their responses. Furthermore, additional analyses of the factors influencing the habits of using and disposing of medications are needed, which could not be conducted in this study due to the small number of participants. Additionally, since the questionnaire was conducted among students of the University of Split School of Medicine, the results do not reflect the knowledge and attitudes of the general population regarding ecopharmacovigilance, and further research is needed to explore this area.

## 5. Conclusions

This study showed that future healthcare professionals have a knowledge foundation about EPV to further build on. One encouraging fact is that they were aware of the subjects’ importance and had a desire to learn more about it. Education strategies about EPV are much needed for both healthcare professionals and the public. One of the first steps towards educating people about EPV should be implementing it into the curriculums of study subjects for healthcare and medicine students. Special emphasis should be placed on EDSP in context of EPV and antibiotic resistance. Future prescribers should have a higher level of knowledge coming into the practice than they have now. Future studies testing current healthcare professionals’ knowledge of EPV would give an even better perspective about the state of things.

## Figures and Tables

**Table 1:** The demographic characteristics of the participants.

	Total	Pharmacy	Medicine	Dental Medicine
	N = 80	N = 27	N = 39	N = 14
Gender				
Male	13 (16.3%)	5 (18.5%)	6 (15.4%)	2 (14.3%)
Female	67 (83.7%)	22 (81.5%)	33 (84.5%)	12 (85.7%)

The data are presented as whole numbers (percentages).

**Table 2:** Students’ responses on listed questions.

	Always	Often	Sometimes	Rarely	Never
12. How often do you check the expiration date of the medications?	17 (21.2%)	20 (25%)	24 (30%)	14 (17.5%)	0
14. How often do you take your medications based on advice from physician/pharmacist?	25 (31.2%)	24 (30%)	12 (15%)	17 (21.2%)	2 (2.5%)
15. Whenever multiple medications are prescribed to me/my family members, I/we use only some of them.	0	3 (3.7%)	7 (8.8%)	23 (28.7%)	47 (58.7%)
16. How often do you practice self-medication for milder diseases like flu or headache?	27 (33.7%)	28 (35%)	16 (20%)	9 (11.3%)	0
17. How often do you buy prescription drugs without a prescription?	1 (1.3%)	2 (2.5%)	6 (7.5%)	11 (13.8%)	60 (75%)

The data are presented as whole numbers (percentages).

**Table 3:** The expiration dates are very clear and legible on the medication dosage forms.

	Pharmacy	Medicine	Dental Medicine	Total	p *
	N = 27	N = 39	N = 14	N = 80	
Yes	24	22	9	55	0.053
	(88.9%)	(56.4%)	(64.3%)	(68.7%)	
No	1	7	3	11	
	(3.7%)	(17.9%)	(21.4%)	(13.8%)	
I don’t know	2	10	2	14	
	(7.4%)	(25.6%)	(14.3%)	(17.5%)	

The data are presented as whole numbers (percentages). * Fischer’s exact test.

**Table 4:** Display of the number and proportion of correct answers by field of study.

Pharmacy		Medicine	Dental Medicine	p *
N = 27		N = 39	N = 14	
1.	24 (88.9%)	27 (69.2%)	7 (50.0%)	0.024
2.	26 (96.3%)	37 (94.9%)	13 (92.9%)	1.000 **
3.	27 (100%)	36 (92.3%)	14 (100.0%)	0.284 **
4.	26 (96.3%)	37 (94.9%)	13 (92.9%)	1.000 **
5.	5 (18.5%)	2 (5.1%)	4 (28.6%)	0.048 **
6.	26 (96.3%)	32 (82.1%)	13 (92.9%)	0.228 **
7.	19 (70.4%)	33 (84.6%)	11 (78.6%)	0.380
8.	18 (66.7%)	33 (84.6%)	12 (85.7%)	0.168
9.	27 (100.0%)	36 (92.3%)	14 (100.0%)	0.285 **
10.	21 (77.8%)	36 (92.3%)	14 (100.0%)	0.083 **
11.	2 (7.4%)	5 (12.8%)	4 (28.6%)	0.704 **

The data are presented as whole numbers (percentages). * Chi-square test. ** Fischer’s exact test.

**Table 5:** Students comments on unused medication at home.

“I think it’s important to have rational prescribing of medications, educate patients about the medication itself, and what to do if they stop taking the medication (e.g., due to side effects) and if the medication expires, what to do with it. This education should involve all healthcare professionals and the media.”
“Unused medications in my household are usually some over-the-counter pain relievers, rarely/never antibiotics or other nonsteroidal anti-inflammatory drugs.”
“Pharmacies should educate people more about disposing of expired medications.”
“There is no consideration of the consequences of accumulating unused medications, nor is there awareness about it.”
“I believe education on this topic is necessary, both for the general population and healthcare professionals, because nobody has clearly informed us about such issues personally.”

Table 5 contains all of the participants’ comments.

## Data Availability

Additional study data are available upon request to the study’s authors.

## Supplementary Materials

The following supporting information can be downloaded at: https://www.mdpi.com/article/10.3390/pharmacy12050146/s1, Knowledge and Attitudes of Biomedical Students Regarding Ecopharmacovigilance.

## References

[1] Turner M.G., Calder W.J., Cumming G.S., Hughes T.P., Jentsch A., LaDeau S.L., Lenton T.M., Shuman B.N., Turetsky M.R., Ratajczak Z. (2020). Climate Change, Ecosystems and Abrupt Change: Science Priorities. Phil. Trans. R. Soc. B.

[2] McFadden I.R., Sendek A., Brosse M., Bach P.M., Baity-Jesi M., Bolliger J., Bollmann K., Brockerhoff E.G., Donati G., Gebert F. (2023). Linking Human Impacts to Community Processes in Terrestrial and Freshwater Ecosystems. Ecol. Lett..

[3] Kar S., Pradhan H., Mohanta G. (2010). Concept of Essential Medicines and Rational Use in Public Health. Indian J. Community Med..

[4] Jose J., Sandra Pinto J., Kotian B., Mathew Thomas A., Narayana Charyulu R. (2020). Comparison of the Regulatory Outline of Ecopharmacovigilance of Pharmaceuticals in Europe, USA, Japan and Australia. Sci. Total Environ..

[5] Daughton C.G., Ruhoy I.S. (2008). The Afterlife of Drugs and the Role of PharmEcovigilance. Drug Saf..

[6] Medhi B., Sewal R. (2012). Ecopharmacovigilance: An Issue Urgently to Be Addressed. Indian J. Pharmacol..

[7] Velo G.P. (2007). Why Ecopharmacovigilance?. Drug Saf..

[8] World Health Organisation (2002). The Importance of Pharmacovigilance: Safety Monitoring of Medicinal Products.

[9] Holm G., Snape J.R., Murray-Smith R., Talbot J., Taylor D., Sörme P. (2013). Implementing Ecopharmacovigilance in Practice: Challenges and Potential Opportunities. Drug Saf..

[10] Li S., Guo J., He B., Zhu Y., Wang J. (2021). Environmental Knowledge, Behaviors, and Attitudes Regarding Caffeine Consumption among Chinese University Students from the Perspective of Ecopharmacovigilance. Environ. Sci. Pollut. Res..

[11] Wang J., Li S., Zhu Y., Guo J., Liu J., He B. (2021). Targeted Eco-Pharmacovigilance as an Optimized Management Strategy for Adverse Effects of Pharmaceuticals in the Environment. Environ. Toxicol. Pharmacol..

[12] European Medicines Agency (2019). Guideline on the Environmental Risk Assessment of Medicinal Products for Human Use; EMA/CHMP/SWP/4447/00 Rev. 1; European Medicines Agency. https://www.ema.europa.eu/en/documents/scientific-guideline/guideline-environmental-risk-assessment-medicinal-products-human-use-revision-1_en.pdf.

[13] Wang J., Hu X. (2014). Ecopharmacovigilance: Current State, Challenges, and Opportunities in China. Indian J. Pharmacol..

[14] Rogowska J., Zimmermann A. (2022). Household Pharmaceutical Waste Disposal as a Global Problem—A Review. Int. J. Environ. Res. Public Health.

[15] Wang J., Zhang M., Li S., He B. (2018). Adapting and Applying Common Methods Used in Pharmacovigilance to the Environment: A Possible Starting Point for the Implementation of Eco-Pharmacovigilance. Environ. Toxicol. Pharmacol..

[16] Sapkota B., Pariatamby A. (2023). Pharmaceutical waste management system-Are the current techniques sustainable, eco-friendly and circular? A review. Waste Manag..

[17] Yu X., Hu X., Li S., Zhang M., Wang J. (2019). Attitudes and Practice Regarding Disposal for Unwanted Medications among Young Adults and Elderly People in China from an Ecopharmacovigilance Perspective. Int. J. Environ. Res. Public Health.

[18] European Commission Health and Food Safety Directorate-General, Pharmaceutical Committee Revised Mandate of the Ad-Hoc Working Group to Focus on the EU Strategic Approach on Pharmaceuticals in the Environment. https://health.ec.europa.eu/system/files/2021-11/wg_pharmaceuticals-environment_mandate_en_0.pdf#:~:text=The%20Pharmaceutical%20Committee%20endorsed%20in%20the%20March%202020,fall%20under%20the%20competence%20of%20the%20Member%20States.

[19] Fens T., Moermond C.T.A., van der Maas P., Dantuma-Wering C., Lestestuiver G.H., Szperl A., Schuiling L.C.M., Hak E., Taxis K. (2024). Reducing Pharmaceuticals in Water, a New Module Integrated in the Pharmacy Game: Evaluating the Module’s Effects on Students’ Knowledge and Attitudes. Pharmacy.

[20] Farmacontaminación—Título Propios—UPV/EHU. https://www.ehu.eus/es/web/graduondokoak/experto-universidad-farmacontaminacion.

[21] Sustainable Drug Discovery—International Master. https://sustainabledrugdiscovery.eu/.

[22] Jha N., Shankar P.R., Palaian S. (2021). Knowledge and Practice on Ecopharmacovigilance and Medicine Storage Amongst Medical and Dental Students in Lalitpur, Nepal. RMHP.

[23] Jonjić D., Vitale K. (2014). Issues around Household Pharmaceutical Waste Disposal through Community Pharmacies in Croatia. Int. J. Clin. Pharm..

[24] Manocha S., Suranagi U.D., Sah R.K., Chandane R.D., Kulhare S., Goyal N., Tanwar K. (2020). Current Disposal Practices of Unused and Expired Medicines Among General Public in Delhi and National Capital Region, India. CDS.

[25] Alnahas F., Yeboah P., Fliedel L., Abdin A.Y., Alhareth K. (2020). Expired Medication: Societal, Regulatory and Ethical Aspects of a Wasted Opportunity. Int. J. Environ. Res. Public Health.

[26] Van Eikenhorst L., Salema N.-E., Anderson C. (2017). A Systematic Review in Select Countries of the Role of the Pharmacist in Consultations and Sales of Non-Prescription Medicines in Community Pharmacy. Res. Soc. Adm. Pharm..

[27] Liu J., Wang J., Hu X. (2017). Knowledge, Perceptions, and Practice of Ecopharmacovigilance among Pharmacy Professionals in China. Environ. Monit. Assess..

[28] Alghadeer S., Al-Arifi M.N. (2021). Community Pharmacists’ Practice, Awareness, and Beliefs about Drug Disposal in Saudi Arabia. Healthcare.

[29] Wang J., Li S., He B. (2020). Chinese Physicians’ Attitudes toward Eco-Directed Sustainable Prescribing from the Perspective of Ecopharmacovigilance: A Cross-Sectional Study. BMJ Open.

[30] Region Stockholm Pharmaceuticals and the Environment. https://janusinfo.se/beslutsstod/lakemedelochmiljo/pharmaceuticalsandenvironment.4.7b57ecc216251fae47487d9a.html.

[31] Jain S., Jain P., Moghe V., Seth V., Upadhyaya P., Abhijit K., Goyal J. (2015). A Systematic Review of Prescription Pattern Monitoring Studies and Their Effectiveness in Promoting Rational Use of Medicines. Perspect. Clin. Res..

[32] Agencija za Lijekove i Medicinske Proizvode (HALMED) Izvješće o Potrošnji Lijekova u Republici Hrvatskoj u 2022. https://www.halmed.hr/Novosti-i-edukacije/Publikacije-i-izvjesca/Izvjesca-o-potrosnji-lijekova/Izvjesce-o-potrosnji-lijekova-u-Republici-Hrvatskoj-u-2022/.

[33] Blanco G., Gómez-Ramírez P., Espín S., Sánchez-Virosta P., Frías Ó., García-Fernández A.J. (2023). Domestic Waste and Wastewaters as Potential Sources of Pharmaceuticals in Nestling White Storks (Ciconia Ciconia). Antibiotics.

[34] Viana P., Meisel L., Lopes A., De Jesus R., Sarmento G., Duarte S., Sepodes B., Fernandes A., Dos Santos M.M.C., Almeida A. (2021). Identification of Antibiotics in Surface-Groundwater. A Tool towards the Ecopharmacovigilance Approach: A Portuguese Case-Study. Antibiotics.

[35] Chen D., Liu S., Zhang M., Li S., Wang J. (2018). Comparison of the Occurrence of Antibiotic Residues in Two Rural Ponds: Implication for Ecopharmacovigilance. Environ. Monit. Assess..

[36] Wang J., He B., Hu X. (2015). Human-Use Antibacterial Residues in the Natural Environment of China: Implication for Ecopharmacovigilance. Environ. Monit. Assess..

[37] Liao M., Wei S., Zhao J., Wang J., Fan G. (2023). Risks of Benzalkonium Chlorides as Emerging Contaminants in the Environment and Possible Control Strategies from the Perspective of Ecopharmacovigilance. Ecotoxicol. Environ. Saf..

[38] Eltaib L., Alanazi S. (2020). Practices and Attitudes Concerning Expiration Date, Unused, and Expired Medication Disposal. Int. J. Med. Sci. Public. Health.

[39] Barroso P.F., Moraes C.G., Falavigna M., Sirtori L.R., Cruz F.H.D., Pons E.D.S. (2021). Users and Health Professionals Perceptions of Medication Labels: A Qualitative Approach. RSD.

[40] Gubae K., Arega Moges T., Agegnew Wondm S., Bayafers Tamene F., Kiflu M., Aschale E., Belachew E.A. (2023). Ecopharmacology: Knowledge, Attitude, and Medication Disposal Practice Among Pharmacy Students. Integr. Pharm. Res. Pract..

[41] Akande-Sholabi W., Olaoye D.Q., Adebisi Y.A. (2023). Drug take-back program: Assessment of knowledge, practices, and barriers to safe disposal of unused medication among healthcare students in a Nigerian university. BMC Med. Educ..

[42] Abahussain E., Waheedi M., Koshy S. (2021). Pharmacy students’ knowledge and practices concerning the storing and disposal of household medication in Saudi Arabia. Curr. Pharm. Teach. Learn..

[43] Bashatah A., Wajid S. (2020). Knowledge and Disposal Practice of Leftover and Expired Medicine: A Cross-Sectional Study from Nursing and Pharmacy Students’ Perspectives. Int. J. Environ. Res. Public Health.

