# Supplementary material for: Future Healthcare Workers and Ecopharmacovigilance: Where Do We Stand?
Source: Pharmacy (Basel). 2024 Sep 26;12(5):146. doi: 10.3390/pharmacy12050146 (PMC11511310; doi:10.3390/pharmacy12050146)
Supplement: Supplementary file 1 [file pharmacy-12-00146-s001.zip › pharmacy-3145584-supplementary.pdf]

Knowledge and Attitudes of Biomedical Students Regarding Ecopharmacovigilance

Dear Participant,

We kindly ask you to participate in this survey for the purposes of a thesis project. The questionnaire assesses your knowledge and attitudes related to ecopharmacovigilance. The research is anonymous, your personal data will be processed electronically, and the researchers will adhere to internal procedures for the protection of personal data. Please note that all the information you provide will be used exclusively for the stated scientific research purposes, and your identity will always remain anonymous. By completing the questionnaire, you will be considered to have given your consent to participate in the research.

Thank you for your cooperation!

I. Age (please enter): ____________

II. Gender (please enter):

III. Study program:
a. Medicine
b. Dental Medicine
c. Pharmacy

IV. Year of study:
a. 1st year
b. 2nd year
c. 3rd year
d. 4th year
e. 5th year
f. 6th year

V. Is someone in your immediate family (father, mother, brother, sister) a healthcare worker?
a. Yes
b. No

VI. Your living arrangement:
a. With family
b. In private accommodation
c. In a student dormitory

VII. If you live with your family, does your family include a child under 2 years old?
a. Yes
b. No

VII. Have you used any medications in the last 6 months?
a. Yes
b. No

Knowledge about Storage and Disposal of Unused and Expired Medications

1. “Medication expiration date” means:
   a. The date on which the medication loses 50% of its potency
   b. The date after which the medication should no longer be used

c. The date after which medications can be used for a maximum of 30 days
d. The date when the medication was manufactured

2. The term 'medication disposal system' refers to:
   a. Returning unused medications to the pharmacy and getting a refund
   b. The correct technique for managing medication waste that does not harm the environment
   c. Removing the medication from its original container
   d. Removing the identification of the medication by removing the labels

3. The term "Medication return system" for expired medications is:
   a. Keeping unused medications for future needs
   b. Buying medications at the pharmacy
   c. Depositing expired medications at pharmacies
   d. Claiming medication costs from insurance companies

4. Improper disposal of medications in unauthorized ways can cause:
   a. Harmful effects on the environment
   b. Accidental poisoning of children
   c. Misuse of medications by vulnerable individuals
   d. All of the above

5. The best method for preventing the hazardous effects of unused medications might be:
   a. Guidelines for consumers on safe medication disposal
   b. Returning medications to the pharmacy before their expiration date
   c. Donating unused medications to family and friends
   d. Burying unused medications in landfills and/or in the backyard

6. The best strategy for preventing the hazardous effects of expired medications might be:
   a. Throwing medications in the trash
   b. Prescribing appropriate quantities and durations
   c. Donating unused medications to family members
   d. Donating unused medications to charitable organizations

7. Who should be responsible for improving knowledge among households about the proper disposal of "unused medications"?
   a. Doctor
   b. Pharmacist
   c. Newspapers (media)
   d. Social media
   e. All of the above

8. The person(s) responsible for improving knowledge in households about the correct disposal of 'expired medications' is (are):
   a. Doctors
   b. Healthcare workers
   c. Nurses
   d. Social media
   e. All of the above

9. Medications should be stored in:
   a. The kitchen cabinet

b. The bathroom cabinet
c. A cool and dry place
d. The freezer

10. Storing excess medications at home can encourage:
    a. Self-medication
    b. Accidental poisoning
    c. Suicide attempts
    d. All of the above

11. The appropriate way(s) to dispose of expired medications at home is (are):
    a. Throwing medications in the sink and thoroughly rinsing
    b. Throwing them into nearby water bodies like a canal
    c. Throwing them in the trash
    d. Burning medications

Section-3: Practices for Disposal of Unused and Expired Medications

12. How often do you check the expiration date of medications?
    a. Always
    b. Often
    c. Sometimes
    d. Rarely
    e. Not applicable (please specify reason)

13. What do you do with any leftover medication you purchased at home?
    a. Throw it in the household waste
    b. Flush unused medications down the toilet
    c. Keep it at home until it expires
    d. Burn medications together with trash
    e. Return medications to the pharmacy

14. How often do you take medications according to the advice of your doctor/pharmacist?
    a. Always
    b. Often
    c. Sometimes
    d. Rarely
    e. Not applicable (please specify reason)

15. When I/my family members are prescribed multiple medications, I/we only use some of them.
    a. Always
    b. Often
    c. Sometimes
    d. Rarely
    e. Never

16. How often do you practice self-medication for minor ailments like fever and headache?
    a. Always
    b. Often
    c. Sometimes

d. Rarely
e. Never

17. How often do you buy medications that require a valid prescription over the counter (OTC)?
    a. Always
    b. Often
    c. Sometimes
    d. Rarely
    e. Never

18. Expiration dates are very clear and readable on the medication forms.
    a. Yes
    b. No
    c. I don't know

19. The following reasons prevent or limit the use of medications by me/my family member:
    a. Forgetfulness
    b. Ineffectiveness of medications
    c. Adverse effects of medications
    d. Improvement of health condition

20. Have you ever educated your friends/family members about the safe disposal of medications?
    a. Yes
    b. No
21. Please provide any other comments you have about unused medications at home.
